# Chronic Monoarthritis Pain Accelerates the Processes of Cognitive Impairment and Increases the NMDAR Subunits NR2B in CA3 of Hippocampus from 5-month-old Transgenic APP/PS1 Mice

**DOI:** 10.3389/fnagi.2017.00123

**Published:** 2017-05-12

**Authors:** Wei-Yi Gong, Rong Wang, Yuan Liu, He Jin, Zhi-Wei Zhao, Yu-Lan Wang, Hong-Yan Li, Xu Zhang, Jia-Xiang Ni

**Affiliations:** ^1^Central Laboratory, Xuanwu Hospital of Capital Medical University, Laboratory for Neurodegenerative Disease of Ministry of Education, Center of Alzheimer’s Disease, Beijing Institute for Brain DisordersBeijing, China; ^2^Department of Pain Management, Xuanwu Hospital, Capital Medical UniversityBeijing, China; ^3^Department of Anesthesiology, Fujian Medical University Union HospitalFuzhou, China

**Keywords:** chronic pain, learning and memory, cognitive impairment, N-methyl-D-aspartic acid receptor (NMDAR), neurotoxicity, hippocampus, mouse model

## Abstract

Many factors impact cognitive impairment; however, the effects of chronic pain and the mechanisms underlying these effects on cognitive impairment are currently unknown. Here we tested the hypothesis that chronic pain accelerates the transition from normal cognition to mild cognitive impairment (MCI) in 5-month-old transgenic APP/PS1 mice, an animal model of Alzheimer’s disease (AD), and that neurotoxicity induced by N-methyl-D-aspartic acid receptor (NMDAR) subunits may be involved in this process. Chronic monoarthritis pain was induced in transgenic APP/PS1 mice and 5-month-old wild-type (WT) mice by intra- and pre-articular injections of Freund’s complete adjuvant (FCA) into one knee joint. Pain behavior, learning and memory function, and the distribution and quantity of NMDAR subunits (NR1, NR2A and NR2B) in hippocampal CA1 and CA3 regions were assessed. Our results showed that although persistent and robust monoarthritis pain was induced by the FCA injections, only the transgenic APP/PS1 mice with chronic monoarthritis pain exhibited marked learning and memory impairments. This result suggested that chronic monoarthritis pain accelerated the cognitive impairment process. Furthermore, only transgenic APP/PS1 mice with chronic monoarthritis pain exhibited an overexpression of NR2B and an increased NR2B/NR2A ratio in the hippocampus CA3. These findings suggest that chronic pain is a risk factor for cognitive impairment and that increased neurotoxicity associated with NMDAR subunit activation may underpin the impairment. Thus, NMDARs may be a therapeutic target for the prevention of chronic pain-induced cognitive impairment.

## Introduction

Mild cognitive impairment (MCI) is dysfunction in memory, learning and attention that is not dementia but is not normal for the given age and does not amount to significant impairments in functional activities (Petersen, 2004). MCI affects 15% of nondemented persons aged more than 70 years (Roberts et al., 2008), and those who have MCI progress to dementia in higher proportions than cognitively normal individuals (Mitchell and Feshki-Shiri, 2009; Petersen et al., 2009). Chronic pain is also common in older people and often coexists with cognitive impairment with or without dementia (Peisah et al., 2014; Sampson et al., 2015; van Kooten et al., 2015). An epidemiological study has shown that approximately 50% of patients with dementia experience pain (Corbett et al., 2012), and pain frequently decreases cognitive performance (Scherder et al., 2008). Although a link between chronic pain and cognitive dysfunction has not been fully clarified, evidence shows that chronic pain in patients with temporomandibular disorder (Moayedi et al., 2012), fibromyalgia (Kuchinad et al., 2007), and back pain (Apkarian et al., 2004) accelerates whole brain gray matter atrophy.

The N-methyl-D-aspartate receptor (NMDAR) is an important ionotropic glutamic acid receptor, and it is formed by combinations of different subunits, including NMDAR1 (NR1), NMDAR2 (NR2A, NR2B, NR2C, NR2D), and NMDAR3 (NR3A, NR3B). These NMDARs subunits exhibit not only differential distribution but also variable functions in the nervous system. Although their normal expression levels and functions are critical for normal functioning of the central nervous system (CNS), abnormalities in NMDARs can cause excitotoxicity leading to subsequent neurodegeneration, as is observed in patients with Alzheimer’s diseases (AD). Recent studies indicate that excessive activation of extrasynaptic NR2B participates in the amyloid (A)β-induced loss of synapses and synaptic proteins in neuronal cell cultures (Liu et al., 2010; Rönicke et al., 2011; Chang et al., 2016). Tau phosphorylates NMDARs, which enhances the downstream neurotoxic effects (Ittner et al., 2010). Peripheral (Du et al., 2003; Chen W. et al., 2014), spinal (Lefèvre et al., 2015; Haley and Dickenson, 2016) and supraspinal (Ghelardini et al., 2008; Wei et al., 2008; Da Silva et al., 2010; Cheng et al., 2011; Ho et al., 2013; Ohsawa et al., 2014) NMDARs together contribute to the development and maintenance of chronic pain, but supraspinal NMDAR overexpression in particular enhances responsiveness to noxious stimulation and aggravates chronic pain conditions. Because abnormal expression levels and altered functions of NMDARs are involved in AD and chronic pain, we hypothesized that chronic pain accelerates the progression of cognitive impairment in patients at high risk for dementia, and that the neurotoxicity induced by NMDAR subunits in the hippocampus may be a mechanistic link between AD and chronic pain. Several previous preclinical studies using inflammatory pain and neuropathic pain models have investigated the effects of pain on cognition, and their results confirm that pain worsens attention (Boyette-Davis et al., 2008; Pais-Vieira et al., 2009), emotional decision-making (Ji et al., 2010) and spatial learning and memory (Leite-Almeida et al., 2009; Hu et al., 2010). However, it is still unclear whether chronic pain alters the processes involved in the progression from normal cognition to dementia. No studies have yet explored the relationship between pain and cognitive impairment by examining the mechanisms of action for the involved NMDAR subunits in transgenic animal models of AD.

Therefore, the aim of the present study was to evaluate the effects of chronic pain on cognitive dysfunction by determining pain behavior and cognitive status of 5-month-old transgenic APP/PS1 mice, which are considered a murine model of AD, subjected to a chronic monoarthritis model of pain. Because localization and subunit composition of NMDARs appear to produce paradoxical actions and because NR1, NR2A and NR2B appear to be particularly important for nociception and AD, we also investigated the distribution and expression of these subunits in the hippocampus.

## Materials and Methods

### Animals

APP/PS1 transgenic mice (C57BL/6J) were supplied by the Institute of Laboratory Animal Science at Peking Union Medical College. These mice express a mouse-human hybrid transgene containing the extracellular and intracellular regions of the mouse sequence and a human sequence within the Aβ domain containing Swedish mutations (K594N/M595L), and they also express the exon 9-deleted variant of human presenilin 1 (Zhang et al., 2011; Zong et al., 2011). The animals were housed individually under standard conditions (23–25°C; humidity 50%–60%; 12-h light/dark cycle with lights on at 8:00 a.m.; *ad libitum* access to water and food). All animals were given health checks and acclimatized for 1 week before the experiment. The study was conducted following the recommendations of the Animal Care and Use Committee at Capital Medical University, and the experimental procedures were approved by the Animal Care and Use Committee at the Capital Medical University.

All experiments were performed in 5-month-old wild-type (WT) male C57BL/6J and congenic male APP/PS1 transgenic mice, also 5 months old. The APP/PS1 transgenic mouse strains have been previously described and characterized in multiple studies (Zhang et al., 2011; Wang D. et al., 2013; Wang D. M. et al., 2013). Experiments were conducted using 24 WT mice and 24 APP/PS1 mice. The animals were divided into the following four treatment groups (*n* = 12 per group): animals injected with Freund’s complete adjuvant (FCA; thus, FCA-WT and FCA-APP groups) and saline-injected animals (Sham-WT and Sham-APP groups). Body weight (expressed in grams), as an important health indicator, was measured before treatment and 1, 7 and 14 days and 1 and 2 months after treatment. Six animals were excluded for failure to achieve spatial acquisition. Thus, body weights, knee joint diameters, pain scores and spatial acquisition and probe test scores were obtained from a total of 42 animals as follows: FAC-APP (*n* = 11), FAC-WT (*n* = 11), Sham-APP (*n* = 10) and Sham-WT (*n* = 10). After completion of the behavioral experiments, immunofluorescence was performed using five animals from each group, and western blotting was performed using the following additional numbers of animals: FAC-APP (*n* = 6), FAC-WT (*n* = 6), sham-APP (*n* = 5) and sham-WT (*n* = 5). All behavioral measurements and morphological evaluations were conducted by an experimenter who was blinded to the experimental groups.

### Induction of Chronic Monoarthritis Pain

Animals were anesthetized using 2%–3% isoflurane mixed with air at a flow rate of 2 L/min. A model of chronic monoarthritis pain was produced by giving one intra-articular injection of FCA (20 μL, 10 mg/mL) and four peri-articular injections of FCA (four injections of 20 μL each) into the right knee joint (Kelso et al., 2007). One month after the first injection, the same knee joint was reinjected with the same protocol to maintain chronic pain. Animals in the sham groups received the same injection protocol but saline was used rather than FCA. The bilateral hind knee joint diameter (expressed in mm) was measured using a vernier caliper before injection and 1, 7 and 14 days and 1 and 2 months after the initial injection.

### Behavioral Measures of Arthritic Joint Pain

Using previously published methods (Ghilardi et al., 2012; Jimenez-Andrade and Mantyh, 2012), behavioral measures of arthritic joint pain, including spontaneous pain (flinching) and stimulus-evoked pain (limb use), were performed before injection and 1, 7 and 14 days and 1 and 2 months after the intital injection. Flinching was defined as the animal spontaneously raising its hind paw in an act representative of spontaneous nocifensive behavior. To evaluate resting pain levels, the number of spontaneous flinches was recorded over a 2-min period. Normal limb use during spontaneous ambulation in an open field apparatus was used as an indicator of stimulus-evoked pain and was scored on a scale of 0–5 as follows: 0, complete lack of limb use; 1, partial non-use of limb during locomotor activity; 2, limping and guarding behavior; 3, pronounced limp; 4, partial but not pronounced limp; 5, normal behavior.

### Morris Water Maze (MWM) Task

The Morris water maze (MWM) was used to assess spatial learning and memory. The apparatus and protocol for the MWM task used in this study were based on those described in a previous report (Vorhees and Williams, 2006). Briefly, the maze consisted of a white circular pool (122 cm in diameter and 51 cm in height) filled with opaque water at a temperature of approximately 22°C. The pool was conceptually divided into four quadrants and had four points designated as starting positions: north, east, southeast and northwest. A white circular platform (10 cm^2^) was submerged 1.0 cm below the water surface and was located in the center of southwest quadrant. The room temperature was constant, the light levels were even, and experimenters remained behind a visual barrier. Monitoring was performed with a video-tracking system (Ethovision XT, Noldus Ltd, Holland).

The spatial acquisition trial was performed as four trials per day for five consecutive days. The animals were released into the water at water-level at one of four starting positions, facing the pool wall. The video-tracking system started recording the moment that the animals were released and stopped when the animals reached the platform within 60 s. Animals that failed to find the platform within the time limit were guided to the platform. All animals were allowed to stay on the platform for 15 s and then performed in the next trial with a 15-min intertrial interval. The starting position changed in each trial. Following the completion of the trials, the mice were dried and returned to their home cages. The time to reach the platform (escape latency), the length of the path taken to find the platform (escape path), and the swimming velocity were measured as indices of learning the spatial task.

Twenty-four hours after the final spatial acquisition trial, a probe trial was performed to assess the animal’s spatial memory. The animals were placed in the water at the northeast starting position, 180° from the original platform position, and swam freely without the platform for 60 s. The number of platform-site crossings, the time spent in the target quadrant, the swimming velocity, and the path length were analyzed.

### Western Blotting

The method reported by Gozal et al. (2002) was followed to conduct western blotting. The animals were anesthetized and their brains rapidly removed and cut into several sections (500 μm/slice) on ice under a surgical microscope. Hippocampal CA1 and CA3 regions were microdissected (Figure 3C) and stored at −80°C until needed. Western blotting was conducted for three proteins, NR1, NR2A and NR2B subunits (Petralia et al., 2010). The hippocampal tissues were homogenized in ice-cold RIPA buffer (0.1% phenylmethylsulfonyl fluoride) and centrifuged at 14,000 *g* for 25 min at 4°C. The protein concentration in the supernatant was determined using a BCA kit (Beyotime Biotechnology, Nanjing, China). Supernatants were mixed with an equal volume of Laemmli buffer and denatured at 100°C for 5 min. Protein samples (40 μg/well) were loaded on 8% polyacrylamide gels according to the manufacterer’s recommendation (Yu Xi Biotechnology Co., Ltd, Jiangyin, China), and then electrophoresed and transferred to nitrocellulose membranes at 4°C. The membranes were then blocked with 5% fat-free milk in Tris-buffered saline containing 0.1% Tween-20 (TBS/T) for 2 h at room temperature and incubated overnight at 4°C with the following primary antibodies at a dilution of 1:1000: mouse monoclonal anti-NR1 (Abcam, Cambridge, UK, ab134308); rabbit monoclonal anti-NR2A, (Abcam, ab133265); and mouse monoclonal anti-NR2B (Abcam, ab93610). The next day, membranes were washed three times for 10 min each in TBST and probed with secondary antibodies diluted 1:1000–2000 (goat anti-mouse IgG or goat anti-rabbit IgG; Santa Cruz, Dallas, TX, USA) for 1 h at room temperature. The membranes were washed again with TBS/T, and immunoreactivity was detected by application of the ECL Plus chemiluminescence reagent for 5 min. Protein levels were quantified by determining their optical densities (with arbitrary units) using ImageJ software (National Institutes of Health, Bethesda, MD, USA) and expressed as a ratio relative to β-actin.

**Figure 1:**
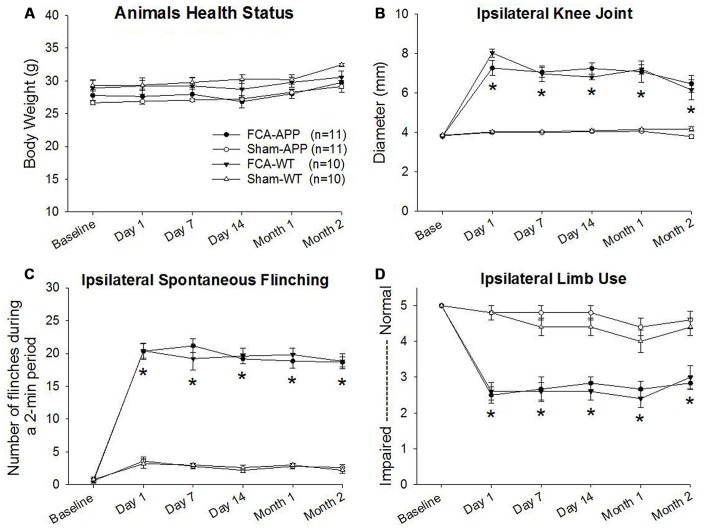
**Significant unilateral knee joint inflammation and pain-related behaviors develop in animals with chronic monoarthritis pain.** Right knee joints of mice were injected twice with a 1-month interval with Freund’s complete adjuvant (FCA) to induce chronic monoarthritis and long-term pain behavior. Saline, instead of FCA, was injected as a control. **(A)** No significant differences are detected in body weights among the mice in the four groups throughout the duration of the experiment. **(B)** Ipsilateral knee joint diameters in FCA-treated animals are significantly greater than those in saline-treated animals, and the swelling remains at significantly higher levels following the initial injection with FCA. **(C)** The number of spontaneous flinches in FCA-treated animals significantly increases following the initial injection and remains at a significantly higher level over time as compared with those in saline-treated animals. **(D)** Ipsilateral hind limb use in FCA-treated animals significantly decreases following the initial injection and remains significantly lower during the experimental period. *P < 0.001 compared with saline-treated animals. Data are presented as the mean ± SEM. FCA-APP (*n* = 11), Sham-APP (*n* = 11), FCA-wild-type (WT) (*n* = 10) and Sham-WT (*n* = 10).

**Figure 2:**
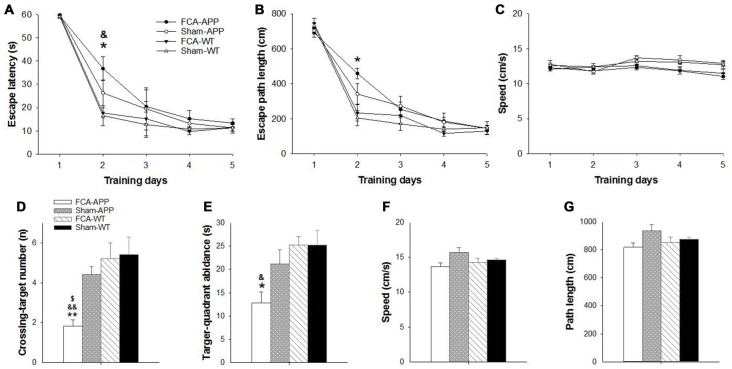
**Significant learning and memory impairments are exhibited in the Morris water maze (MWM) task by APP/PS1 transgenic animals with chronic monoarthritis pain.** Animals were trained to find the platform under the water surface in spatial acquisition trials **(A–C)** and then tested in a probe trial **(D–G)** without the platform 24 h after the last training trial. **(A)** FCA-treated APP transgenic animals show a greater escape latency than FCA-treated and saline-treated WT animals on the second day. **(B)** The FCA-treated APP transgenic animals show a longer escape path length than saline-treated WT animals on the second day. **(C)** No significant difference in swimming velocity is detected among the four groups. **(D)** The FCA-treated APP transgenic animals cross the platform site in the target quadrant fewer times than the saline-treated transgenic animals and the FCA-treated and saline-treated WT animals. **(E)** The FCA-treated APP transgenic animals spend less time in the target quadrant than the FCA-treated and saline-treated WT animals. **(F,G)** No significant difference is detected in swimming velocity and path length traveled among the four groups. *P < 0.05, **P < 0.001 for FCA-APP animals compared with Sham-WT; ^&^*P* < 0.05, ^&&^*P* < 0.01 for FCA-APP animals compared with FCA-WT; ^$^*P* < 0.05 for FCA-APP animals compared with Sham-APP. Data are presented as the mean ± SEM. FCA-APP (*n* = 11), Sham-APP (*n* = 11), FCA-WT (*n* = 10) and Sham-WT (*n* = 10).

**Figure 3:**
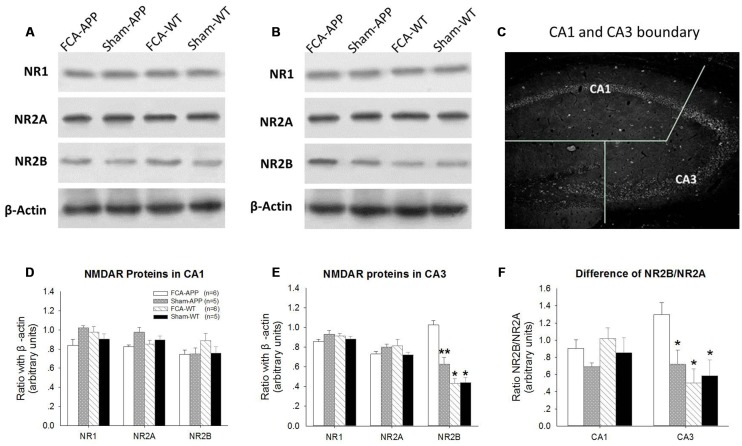
**NR2B protein and ratio of NR2B/NR2A significantly increase in CA3 tissue obtained from APP/PS1 transgenic animals with chronic monoarthritis pain.** Western blotting analysis was performed to quantify NR1, NR2A and NR2B protein expression in hippocampus CA1 and CA3 of APP/PS1 transgenic animals and WT animals with or without chronic monoarthritis pain. **(A)** Representative images of NR1, NR2A, NR2B and β-actin in CA1. **(B)** Representative images of NR1, NR2A, NR2B and β-actin in CA3. **(C)** Schematic illustration of the dissection procedure used for regional analyses. **(D,E)** Quantitative analysis revealed no significant difference in NR1, NR2A and NR2B protein expression in CA1 among the four groups. **(E)** Quantitative analysis revealed no significant difference in NR1 and NR2A in the CA3 among the four groups, but markedly increased NR2B protein expression in CA3 of APP/PS1 transgenic animals with chronic monoarthritis pain. **(F)** No significant difference is detected in the ratio of NR2B/NR2A in the CA1 among the four groups, but the NR2B/NR2A ratio in the CA3 of APP/PS1 transgenic animals with chronic monoarthritis pain markedly increases. *p < 0.001, **p < 0.05 compared to FCA-APP. Data are presented as the mean ± SEM. FCA-APP (*n* = 6), Sham-APP (*n* = 6), FCA-WT (*n* = 5) and Sham-WT (*n* = 5).

### Immunofluorescence and Imaging

Animals were anesthetized with sodium pentobarbital (50 mg/kg, Sigma-Aldrich, St. Louis, MO, USA) at the end of the experiment, and perfused intracardially with 20 mL of 0.1 M phosphate-buffered saline (PBS; pH 7.4 at 4°C) followed by 4% paraformaldehyde (pH 7.4) for fixation. The brains were harvested, post-fixed, and then embedded in paraffin.

Paraffin-embedded tissues were cut into 4-μm-thick sections and collected on polylysine-coated slides. All slides were processed on the same day and were exposed to freshly made reagents at the same time to minimize variations in conditions among groups. The sections were deparaffinized in xylene, rehydrated in a graded series of ethanol, and rinsed with running cold tap water. After antigen retrieval was performed using citrate buffer (0.01 mol/L, pH 6.0) for 15 min in a microwave oven, the sections were blocked with 10% normal goat serum in 0.1% Triton X-100, and then incubated with a primary antibody to mouse monoclonal anti-NR1 (diluted 1:200; Abcam, ab134308) in a humidified chamber at 4°C overnight. For double immunostaining, sections were simultaneously incubated with primary antibodies to rabbit monoclonal anti-NR2A (diluted 1:200; Sigma-Aldrich, St. Louis, MO, USA, SAB4501304) and mouse monoclonal anti-NR2B (diluted 1:100; Abcam, ab93610).

The following day, these slides were rinsed and incubated with a secondary antibody (goat anti-mouse IgG labeled with Alexa Fluor 488 for NR1) for 2 h at room temperature. For double immunostaining, sections were rinsed and simultaneously incubated with secondary antibodies (goat anti-rabbit IgG labeled with Alexa Fluor 594 for NR2A, and goat anti-mouse IgG labeled with Alexa Fluor 488 for NR2B; both diluted 1:200 in TBS/T). After being rinsed three times in PBS for 5 min each, all sections were mounted with Vectashield mounting medium containing DAPI (Vector Laboratories, Burlingame, CA, USA). The primary antibody incubation was omitted in one section as a negative control.

All sections were viewed under a Nikon Eclipse 80i microscope (Nikon Instruments INC, Tokyo, Japan), and images were captured using a Nikon D40 camera system (Nikon Corporation, Tokyo, Japan) using Northern Eclipse software (Empix Imaging, Mississauga, ON, Canada) at magnifications of 10×, 20× and 40× in three channels, blue DAPI (cell nuclei), green Alexa Fluor 488 (NR1 or NR2B), and red Alexa Fluor 594 (NR2A). The photomicrographs were saved as TIFs and quantitatively analyzed using ImageJ software. The mean immunofluorescence intensities of NR1, NR2A and NR2B in hippocampal CA1 and CA3 regions were measured by one experimenter blinded to the conditions to ensure consistency. The CA1 and CA3 boundary was determined based on the method reported by Gozal et al. (2002); Figure 3C.

### Data Presentation and Statistical Evaluations

Analysis of the data was performed using SPSS Version 22.0 (SPSS Inc., Chicago, IL, USA). All data were tested using the Lilliefors correction to the Kolmogorov-Smirnov test for normal distribution and by Levene’s test for homogeneity of variance. After the means were compared using one-way analysis of variance (ANOVA), *post hoc* tests using Bonferroni’s correction were performed for detection of the differences among groups. A value of *P* < 0.05 was considered statistically significant. Data are presented as the mean ± SEM.

## Results

Progressive and stable body weights were exhibited throughout the duration of the experiment, and there were no significant differences in body weights among the four groups (*P* > 0.05), indicating that all animals were healthy (Figure 1A).

### Knee Joint Inflammatory and Pain Behaviors

Significant ipsilateral knee joint inflammation and pain-related behaviors developed in the groups of mice subjected to the chronic monoarthritis pain model protocol. Compared with those in saline-treated animals, the ipsilateral knee joint diameters of FCA-treated animals increased significantly after the initial injection throughout the period of the experiment (*P* < 0.001; Figure 1B). No significant change with time was detected in the contralateral knee joint (*P* > 0.05). Pain-related behaviors, including spontaneous pain (flinching) and evoked pain (limb use), were assessed throughout the duration of the experiment. FCA-treated animals exhibited a greater number of ipsilateral spontaneous flinches than saline-treated animals after the initial injection (*P* < 0.001, Figure 1C). The evaluation of ambulatory pain revealed significantly reduced ipsilateral limb use scores in FCA-treated animals compared with those in saline-treated animals (*P* < 0.001, Figure 1D). By contrast, the contralateral knee joint presented no significant differences in spontaneous flinches and limb use between these four groups (*P* > 0.05). Together, these data indicated that the mice subjected to the chronic monoarthritis pain protocol showed consistent and robust ipsilateral pain for the 2-month duration of this study.

### Morris Water Maze Test

The MWM task was used to examine spatial learning and memory 2 months after chronic monoarthritis pain was induced. We found significant learning and memory impairments in the APP/PS1 transgenic animals with chronic monoarthritis pain.

During the spatial acquisition trials, all animals exhibited similar escape latencies (Figure 2A) and length of escape paths (Figure 2B), and there was no significant difference in swimming velocity among the four groups (Figure 2C). However, the APP/PS1 transgenic animals with chronic monoarthritis pain took more time to locate the platform compared with the WT animals both with and without chronic monoarthritis pain (Figure 2A) and traveled a longer path on the second day compared with the WT animals without chronic monoarthritis pain (Figure 2B). In the probe trial, the APP/PS1 transgenic animals with chronic monoarthritis pain crossed the platform site fewer times (Figure 2D) and spent less time in the target quadrant (Figure 2E) than animals in the other three groups, but there was no significant difference in swimming velocity and path length among the four groups (Figures 2F,G).

### Western Blotting Analysis

We found that NR2B protein expression was significantly increased in hippocampal CA3 tissue obtained from APP/PS1 transgenic animals with chronic monoarthritis pain. Protein expression levels of NR1, NR2A and NR2B in the CA1 (Figures 3A,D) and CA3 areas (Figures 3B,E) were measured by western blotting analysis to investigate the response of NMDARs in APP/PS1 transgenic and WT animals with and without chronic monoarthritis pain. The ratios of NR2B/NR2A were also calculated (Figure 3F). As also shown in the representative images of the CA1 NR1, NR2A and NR2B western blots (Figure 3A), the quantification results indicated that there was no significant difference among the four groups for CA1 region NR1, NR2A and NR2B protein expression (*P* > 0.05, Figure 3D). Similarly, there was no significant difference among the four groups in NR1 and NR2A protein expression levels in the CA3 area (*P* > 0.05, Figures 3B,E). However, the NR2B protein expression levels in the CA3 area of APP/PS1 animals with chronic monoarthritis pain were significantly higher than those from APP/PS1 animals without chronic monoarthritis pain and from WT animals with or without chronic monoarthritis pain (*P* < 0.05, Figure 3E). No significant difference was found in the ratio of NR2B/NR2A in the CA1 area among the four groups (*P* > 0.05, Figure 3F). However, the NR2B/NR2A ratio in the CA3 of APP/PS1 animals with chronic monoarthritis pain was significantly higher than that from APP/PS1 animals without chronic monoarthritis pain and WT animals with or without chronic monoarthritis pain (*P* < 0.05, Figure 3F).

### Immunofluorescence and Photo-micrograph Analysis

**Figure 4:**
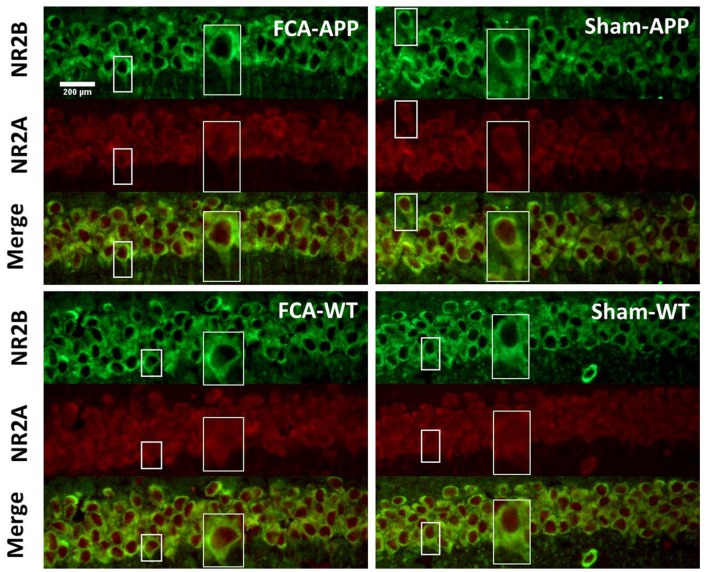
**Representative images of NR2A and NR2B colocalization in the CA1 of the hippocampus (40×).** Green fluorescence indicates NR2B-positive neurons, and red fluorescence indicates NR2A-positive neurons; yellow fluorescence represents the colocalization of NR2A and NR2B after both images are merged. NR2B is located only on the membrane of neurons, but NR2A is diffusely expressed throughout the entire neuron, including the membrane and interior. Thus, NR2A and NR2B are colocalized on neuronal membranes. Scale bars, 200 μm.

**Figure 5:**
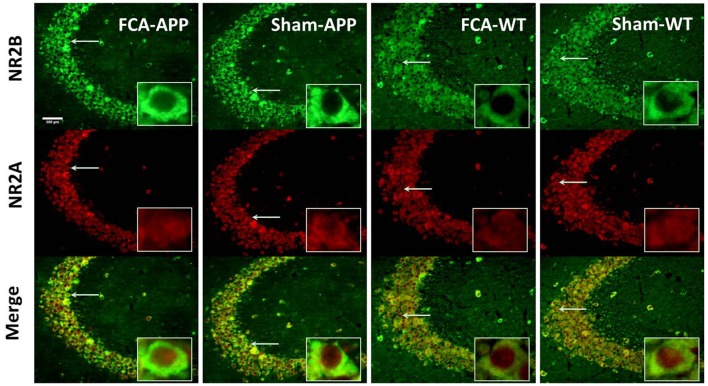
**Representative images of NR2A and NR2B colocalization in hippocampus CA3 (20×).** Green fluorescence indicates NR2B-positive neurons, and red fluorescence indicates NR2A-positive neurons; yellow fluorescence represents the colocalization of NR2A and NR2B after both images are merged. NR2B is located only on the membrane of neurons, whereas NR2A is diffusely expressed throughout the entire neuron, including the membrane and interior; thus, NR2A and NR2B are colocalized on the membranes of neurons. Scale bars, 500 μm.

**Figure 6:**
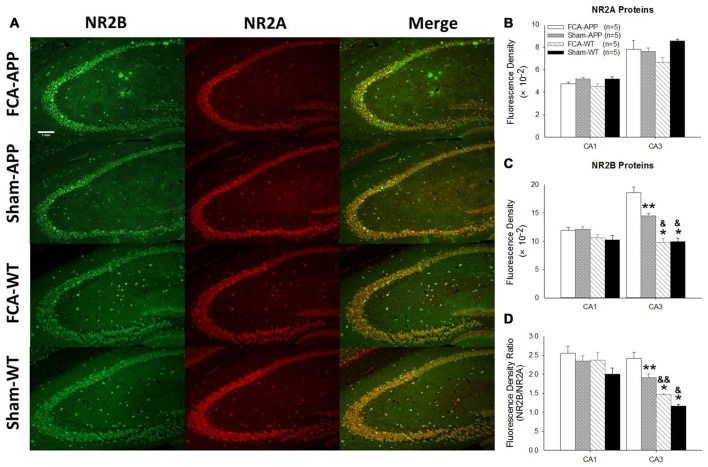
**The mean immunofluorescence intensity of NR2B significantly increases in CA3 tissue of APP/PS1 transgenic animals with chronic monoarthritis pain.** Representative images of NR2A and NR2B in the hippocampus at 10×. **(A)** Quantitative analysis of NR2A and NR2B densities in CA1 and CA3 **(B,C)** and the ratio of NR2B/NR2A **(D)**. **(A)** Green fluorescence indicates NR2B-positive neurons and red fluorescence indicates NR2A-positive neurons; yellow fluorescence represents the colocalization of NR2A and NR2B. **(B)** Quantitative analysis reveals no significant difference in NR2A fluorescence intensity in the CA1 and CA3 among the four groups. **(C)** Quantitative analysis reveals no significant difference in NR2B fluorescence intensity in the CA1 among the four groups. However, in CA3, the NR2B fluorescence intensity in APP/PS1 transgenic animals with chronic monoarthritis pain is the highest of all groups, while that in APP/PS1 transgenic animals without chronic monoarthritis pain was higher than that in WT animals with or without chronic monoarthritis pain. **(D)** Calculations reveal no significant difference in the NR2B/NR2A ratio in the CA1 among the four groups. However, the NR2B/NR2A ratio in the CA3 of APP/PS1 transgenic animals with chronic monoarthritis pain was the highest among all groups, and the ratio in APP/PS1 transgenic animals without chronic monoarthritis pain was higher than that in WT animals with and without chronic monoarthritis pain. *P < 0.001, **P < 0.05 compared with FCA-APP; ^&^*P* < 0.01, ^&&^*P* < 0.05 compared with Sham-APP. Data are presented as the mean ± SEM; *n* = 5. Scale bars, 1 mm.

**Figure 7:**
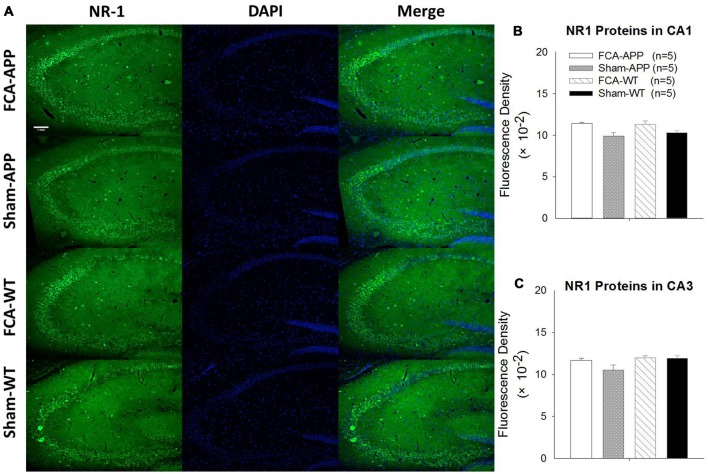
**No qualitative differences are detected in the NR1 expression of the hippocampus CA1 and CA3 regions among the four groups.** Representative images at a magnification of 10× **(A)**, and quantitative analysis of NR1 immunofluorescence signal intensities in the CA1 and CA3 **(B,C)**. **(A)** Green fluorescence indicates NR1-positive neurons, and blue (DAPI) fluorescence indicates cell nuclei. Quantitative analysis reveals no significant difference in NR1 fluorescence signal intensity in the **(B)** CA1 or **(C)** CA3 among the four groups. Data are presented as the mean ± SEM; *n* = 5. Scale bars, 1 mm.

Immunoreactivity of NR1, NR2A and NR2B in the hippocampal CA1 and CA3 areas was assessed using immunofluorescence, and photomicrographs were quantitatively analyzed to determine any differences in NR1, NR2A and NR2B levels or distribution. Figures 4, 5, 6A show images of double immunofluorescence staining for NR2A and NR2B alone or colocalized in merged images captured in the CA1 and CA3. Figure 7A shows the images of NR1 and DAPI-labeled cell nuclei in the CA1 and CA3.

The immunohistochemical results indicated that NR2A and NR2B were colocalized in the CA1 area (Figure 4). While NR2B was located on neuronal membranes, NR2A was expressed not only on the membrane but also in the interior of neurons. Figure 5 shows representative images of NR2A and NR2B double immunostaining in the CA3 area. The distribution of NR2A and NR2B in the CA3 was the same as that in the CA1.

The representative images and quantitative analysis results for NR2A and NR2B are shown in Figure 6. NR2A and NR2B were double-immunostained in CA1 and CA3 neurons (Figure 6A). No qualitative differences were noted among the four groups for NR2A in the CA1 and CA3 (Figure 6B), NR2B in the CA1 (Figure 6C) and the fluorescence density ratio of NR2B/NR2A in the CA1 (Figure 6D). However, the immunofluorescence signal intensity of NR2B (Figure 6C) and the fluorescence density ratio of NR2B/NR2A (Figure 6D) in the CA3 of APP/PS1 transgenic animals with chronic monoarthritis pain were higher than those in any other group, and those in the APP/PS1 transgenic animals without chronic monoarthritis pain were higher than those from WT animals with or without chronic monoarthritis pain. The representative images and quantitative analysis of NR1 are shown in Figure 7. NR1 was widely distributed on neurons in both the CA1 and CA3 regions (Figure 7A), and no qualitative differences were noted in these regions among the four groups (Figures 7B,C).

## Discussion

Transgenic APP/PS1 mice on a C57BL/6J background at 5 months age were used in this study because these animals naturally develop learning and memory impairments as assessed in the MWM task until 12 months of age (Lalonde et al., 2005; Volianskis et al., 2010). The cognitive impairment exhibited in the MWM is unrelated to locomotor effects because learning and memory performances are insensitive to swimming velocity (Fitzgerald and Dokla, 1989). Mice of the C57BL/6J strain have been used to developed a model of robust chronic monoarthritis, as described in a previous publication (2-Kelso et al., 2007), with pain behaviors demonstrating a unilaterally painful condition. Thus, transgenic APP/PS1 and congenic C57BL/6J mice subjected or not to a model of chronic monoarthritis pain were well suited for use to test our hypothesis.

### Chronic Monoarthritis Pain Accelerates the Transition from Normal Cognition to Mild Cognitive Impairment

Chronic pain is a persistently negative condition, and its impacts are far-reaching in every way, including emotional changes and cognitive deficits. Especially in elderly patients, chronic pain frequently coexists with cognitive impairment, and patients experiencing pain display pain-related working memory deficits (Buckalew et al., 2008, 2010). Patients with chronic back pain and complex regional pain syndrome also show significantly less bilateral hippocampal volume (Mutso et al., 2012). Although the cerebral cortex and medial temporal lobe play key roles in memory and attention, the hippocampus is essential for the transference of short- to long-term memory and in the control of spatial memory.

The MWM task used in the current study is a valid mean for measuring hippocampus-dependent spatial navigation and reference memory (Morris, 1984). Our results using this task demonstrated that chronic monoarthritis pain impaired the hippocampal-related learning and memory function of APP/PS1 transgenic mice at 5 months of age. This is consistent with other studies that have shown that pain-induced structural and functional abnormalities in the hippocampus contribute to cognitive impairment. A study by Arai et al. (2013) showed that peripheral pain significantly decreases rat hippocampal miRNA. Animals with neuropathic pain also exhibit reduced hippocampal neurogenesis and altered short-term synaptic plasticity (Mutso et al., 2012), deficits in long-term potentiation (LTP; Kodama et al., 2011) and impaired enriched-environment neurogenesis (Terada et al., 2008).

### Chronic Monoarthritis Pain Increases the Expression of NR2B in the Hippocampal CA3 Region of APP/PS1 Transgenic Mice Aged 5 Months

It is generally agreed that tissue injury contributes to the hyperalgesia associated with nociceptor sensitization and central sensitization by peripheral and spinal activation of NMDARs. Recent evidence has further revealed that chronic pain induces the overexpression of NMDARs in the rostral ventromedial medulla (Wei et al., 2008), the midbrain ventrolateral periaqueductal gray (Ho et al., 2013), and the anterior cingulate cortex (Wu et al., 2005), which then contributes to the development and maintenance of hyperalgesia. However, the effect of pain on the expression of NMDARs in the hippocampus remains controverial. Hippocampal NR2B protein levels significantly increase in irritable bowel syndrome-like rats, a model of chronic visceral pain (Chen Y. et al., 2014). Postoperative pain upregulates the levels of NMDAR2 subunits in the hippocampus (Chi et al., 2013). By contrast, the partial sciatic nerve ligation model of neuropathic pain reduces the expression of NR1 and NR2B in the hippocampus of injured rats (Wang et al., 2015). Our results provide the first evidence that chronic pain induces the overexpression of NR2B in the CA3 of hippocampus in transgenic APP/PS1 mice.

### Chronic Pain Accelerates the Transition from Normal Cognition to Mild Cognitive Impairment through NR2B-Induced Neurotoxicity in the CA3

The results of our study indicated that chronic pain may have provoked the neurotoxic effects of NR2B in the hippocampus and that this neurotoxicity may be responsible for the impaired learning and memory exhibited in APP/PS1 transgenic mice. Chi et al. (2013) also determined that postoperative pain upregulates the levels of NMDAR2 subunits in the hippocampus and contributes to the development of memory deficits. However, the effects of NMDAR are complex and can promote either neuroprotection or excitotoxicity. The distinct subunit expression, trafficking and localization of NMDARs also lead to different activity levels and functions in neurons. Two models, the localization model and the subunit composition model, explain the neurotrophic and excitotoxic effects of NMDARs. Activation of extrasynaptic NMDARs contributes to neurotoxic effects, whereas activation of synaptic NMDARs leads to neuroprotective effects (Hardingham et al., 2002; Hardingham and Bading, 2010). Activation of NR2B is excitotoxic, while activation of NR2A is neurotrophic (Liu et al., 2007; Terasaki et al., 2010; Lai et al., 2011). Furthermore, NR2As are largely concentrated within synapses, while NR2Bs are largely extrasynaptic (Tovar and Westbrook, 1999; Traynelis et al., 2010). Thus, the chronic pain-induced increases observed in the present study in NR2B expression (and presumably function, though this was not directly explored in the present study) could mediate excessive Ca^2+^ influx into the neuron and result in a mitochondrial overload of Ca^2+^, which would promote dendritic and synaptic damage, cell necrosis or apoptosis. Through this mechanism, the APP/PS1 transgenic mice subjected to chronic monoarthritis pain may have exhibited poor MWM performance.

Other factors upregulating NR2Bs in the hippocampus have been shown to induce cognitive impairment. Chronic early postnatal scream sound stress upregulates NR2B levels in the CA1 and CA3, downregulates the NR2A/NR2B ratio, and impairs spatial learning and memory in male mice (Hu et al., 2016). Social isolation stress exacerbates aggressive behaviors and increases NR2A and NR2B levels in the hippocampus (Chang et al., 2015). The activation of extrasynaptic NR2B is involved in the Aβ-induced impairment of LTP in hippocampal slices (Ondrejcak et al., 2010; Li et al., 2011; Rönicke et al., 2011). NR2B antagonists rescue the impairment of LTP (Li et al., 2011; Rönicke et al., 2011), loss of synapses and synaptic proteins (Liu et al., 2010; Rönicke et al., 2011) and facilitation of long-term depression (Li et al., 2009) induced by Aβ. But a contradictory result was found using chronic visceral pain: the higher hippocampal NR2B protein levels induced in irritable bowel syndrome-like rats facilitate the LTP of CA1 via tyrosine phosphorylation (Chen Y. et al., 2014). Thus, many unsolved questions will need to be further explored in future studies.

## Study Limitations and Future Directions

Some limitations existed in the preliminary study. First, pathology, including amyloid plaque deposition and neurofibrillary tangle formation, may be modified when animals are subjected to chronic pain. Thus, we will explore the effects of chronic pain, analgesia and NMDAR blockers on pathology in our next study. Second, although a previous study failed to show the superiority of memantine in their sample of patients having moderate-to-severe AD with significant baseline agitation and aggression (Herrmann et al., 2013), we will apply methods in a future study to block NMDARs and examine their effects on cognitive impairment and the NR2B response induced by chronic monoarthritis pain. Third, because we did not distinguish synaptic from extrasynaptic NMDARs in this study, we will also examine this in future studies.

## Conclusion

The results of this study demonstrated that chronic monoarthritis pain accelerated the appearance of impaired spatial learning and memory in a transgenic mouse model of AD, supporting our hypothesis that chronic pain accelerates cognitive impairment processes. The levels of the NR2B NMDAR subunit in the hippocampal CA3 region increased most in transgenic mice modeling AD and subjected to chronic monoarthritis pain. We speculated that the neurotoxicity generated by this NMDAR subunit induced by chronic pain is responsible for the cognitive impairment observed in the current study. Our results suggest that sufficient analgesia is essential for patients with dementia or at risk of dementia. The exact mechanisms underlying the NMDAR involvement in the cognitive impairment induced by chronic pain warrants further investigation and may provide insight into preventive strategies and therapeutic targets for MCI associated with chronic pain.

## Author Contributions

W-YG contributed to the experimental design, established the animal model, interpreted the data and prepared the manuscript. RW co-designed the study, interpreted the data, prepared the manuscript and provided scientific leadership. YL performed the behavioral experiments, including evaluation of the pain behaviors and the Morris water maze task. HJ assisted in performing the behavioral experiments and establishing the animal model. Z-WZ and Y-LW performed the immunofluorescence and western blot experiments. H-YL and XZ captured the images and analyzed the immunofluorescence and western blot images. J-XN co-designed the experiments and provided practical organization of the study.

## Funding

This study was funded by the Fujian Health Young Cultivation Project Foundation (grant no. 2015-ZQN-JC-15), the Beijing Postdoctoral Research Foundation, and the National Natural Science Foundation of China (grant no. 81472007), and the Capital Health Research and Development of Special (grant no. 2014-1-1031).

## Conflict of Interest Statement

The authors declare that the research was conducted in the absence of any commercial or financial relationships that could be construed as a potential conflict of interest.
